# Resilience in patients and family caregivers living with congenital disorders of glycosylation (CDG): a quantitative study using the brief resilience coping scale (BRCS)

**DOI:** 10.1186/s13023-024-03043-x

**Published:** 2024-03-04

**Authors:** Joana Poejo, Ana Isabel Gomes, Pedro Granjo, Vanessa dos Reis Ferreira

**Affiliations:** 1 CDG & Allies - Professionals and Patient Associations International Network (CDG & Allies-PPAIN), Department of Life Sciences, School of Science and Technology, Universidade NOVA de Lisboa, 2819-516 Caparica, Portugal; 2 Centro de Investigação Em Ciência Psicológica (CICPSI), Faculdade de Psicologia, Universidade de Lisboa, Alameda da Universidade, 1649-013 Lisbon, Portugal; 3 UCIBIO - Applied Molecular Biosciences Unit, Department of Life Sciences, NOVA School of Science and Technology, Universidade NOVA de Lisboa, 2819-516 Caparica, Portugal; 4 Associate Laboratory i4HB - Institute for Health and Bioeconomy, NOVA School of Science and Technology, Universidade NOVA de Lisboa, Caparica, Portugal; 5 Portuguese Association for Congenital Disorders of Glycosylation (CDG), Department of Life Sciences, NOVA School of Science and Technology, Universidade NOVA de Lisboa, 2819-516 Caparica, Portugal

**Keywords:** Resilience, Congenital disorders of glycosylation (CDG), Rare diseases, Brief resilience coping scale (BRCS), Mental health

## Abstract

**Background:**

Patients and family caregivers living with Congenital Disorders of Glycosylation (CDG) experience a heavy burden, which can impact their resiliency and quality of life. The study’s purpose was to measure the resilience levels of patients and family caregivers living with CDG using the brief resilience coping scale.

**Methods:**

We conducted an observational, cross-sectional study with 23 patients and 151 family caregivers living with CDG. Descriptive analyses were performed to characterize patients with CDG and family caregivers’ samples. Additionally, we assessed correlations between resilience and specific variables (e.g., age, academic degree, time until diagnosis) and examined resilience differences between groups (e.g., sex, marital status, occupation, professional and social support).

**Results:**

GNE myopathy was the most prevalent CDG among patients, while in family caregivers was PMM2-CDG. Both samples showed medium levels of resilience coping scores. Individuals with GNE myopathy had significantly higher scores of resilience compared to patients with other CDG. Resilience was positively correlated with educational degree in patients with CDG. Family caregivers had marginally significant higher scores of resilience coping if they received any kind of professional support or had contact with other families or people with the same or similar disease, compared with unsupported individuals.

**Conclusions:**

Despite the inherited difficulties of living with a life-threatening disease like CDG, patients and family caregivers showed medium resilient coping levels. Resilience scores changed significantly considering the CDG genotype, individual's academic degree and professional and social support. These exploratory findings can empower the healthcare system and private institutions by promoting the development of targeted interventions to enhance individuals` coping skills and improve the overall well-being and mental health of the CDG community.

## Background

The concept of resilience arose in 1970, and since then, numerous studies have proliferated in the field of psychology and clinical medicine [1–6]. Resilience comprises a two-step process: i) exposure to significant life adversities and ii) overcoming the challenges to achieve positive outcomes [7]. Therefore, resilience represents a mechanism of coping and rising by adapting to changes, confronting negative stressors, and avoiding the manifestation of significant dysfunctions [8]. Research has proved that resilience is key to individuals‘ mental health, well-being, and quality of life (QoL) [9–11].

Patients facing a chronic or rare disease often suffer from mental health conditions, like depression and anxiety, at higher rates than the general population [12, 13]. This may impact resilience levels and decrease the health-related quality of life (HRQoL) across social, physical, and psychological domains [14–20]. In recent years, studies have also highlighted the role of resilience in managing chronic diseases. High levels of resilience could prevent disease onset and promote health, accelerate healing, facilitate a productive life, and enhance overall well-being despite chronic illness [21, 22].

A family caregiver is a family member, relative, or friend who provides unpaid support for a person with a chronic or disabling condition [23]. They are vital elements in providing complex healthcare tasks (from basic daily living activities to monitoring medical treatment) in addition to social, psychological, emotional, and financial support [24]. Caring for someone can be associated with personal satisfaction in relieving another’s discomfort, feeling useful, and finding meaning in life [25]. However, family caregivers found their situations extremely demanding, which causes a heavy care burden affecting their personal QoL and consequently increasing the risk of developing mental health issues [26–29]. By adopting a resilient coping style, family caregivers can overcome stressful conditions, cope with complex challenges, and adjust to the negative impact, reducing the burden and emotional distress [30].

Congenital disorders of glycosylation (CDG) belong to a group of rare diseases characterized by genetic defects in glycoprotein and glycolipid glycans modification pathways [31]. The first CDG was reported in 1980 [32] and, since then, the number of CDG have increased exponentially, with 163 CDG discovered so far [33]. The assortment and complexity of genetic and clinical features as the multi-organ impairment, even among patients with the same CDG, cause high heterogeneity in this group of rare diseases [34]. The most common symptoms are related to the central nervous system (e.g., neurological impairment and motor disabilities) [35]. Still, other organs (e.g., eyes) [36] and systems (e.g., hepatic, cardiovascular and immune) [37–39] can be affected, causing severe problems. Factors like the complex nature of CDG, severity of symptoms, lack of information, early onset, delay until a final diagnosis [40], the small number of approved therapies [41] restricted to symptom management [42, 43] and the lack of support from informed healthcare and educational providers [44] can cause emotional distress in CDG patients and family caregivers.

In the last two decades, numerous scales were developed to measure resilience levels in different contexts for a specific population, intervention, or outcomes based on different components [45]. The Brief Resilience Coping Scale (BRCS) has been applied to different samples, including the general population [46, 47], family caregivers [48, 49], and persons living with chronic diseases [18, 19, 50, 51]. The lack of published studies on resilience in rare diseases [15] emphasizes the significant gap in research. This oversight can be useful to map indicators of overall psychological adjustment and inform the development of effective interventions to increase resilient coping in vulnerable communities.

The major purpose of this research was to measure the resilience levels of patients and family caregivers living with CDG using the BRCS. Additionally, we aimed to explore associations between resilience and specific variables (e.g., age, academic degree, time until diagnosis) and resilience differences between groups (e.g., sex, marital status, occupation, professional and social support) in both samples. As far as we know, this is the first study evaluating the resilience scores of patients and family caregivers living with CDG. This study sought to provide researchers with deeper insights to help people living with CDG to develop specific coping strategies to address the complex demands of facing the illness burden. Likewise, we intend to raise awareness for mental health issues and create opportunities to advance qualitative research in rare diseases.

## Methods

### Study design

This study followed an observational, cross-sectional design with descriptive and analytical purposes. It is part of a major research (CDG Journey Mapping) that aimed to characterize the CDG community regarding their disease experiences (e.g., physical symptoms and diagnosis onset, informational needs, support and resources available to the CDG community, and identification of gaps on professional and patients’ group support). The CDG Journey Mapping Questionnaire was composed of two distinct survey versions: one for individuals with CDG, their family members, and/or caregivers, and another for professionals such as healthcare providers and researchers. In this study, we concentrated on patients’ and family caregivers’ answers from different sections, with the main emphasis on the "CDG Skills" questionnaire section. The questionnaire was available in English, Spanish, Portuguese, and Italian languages.

### Participants and eligibility criteria

**Fig. 1:**
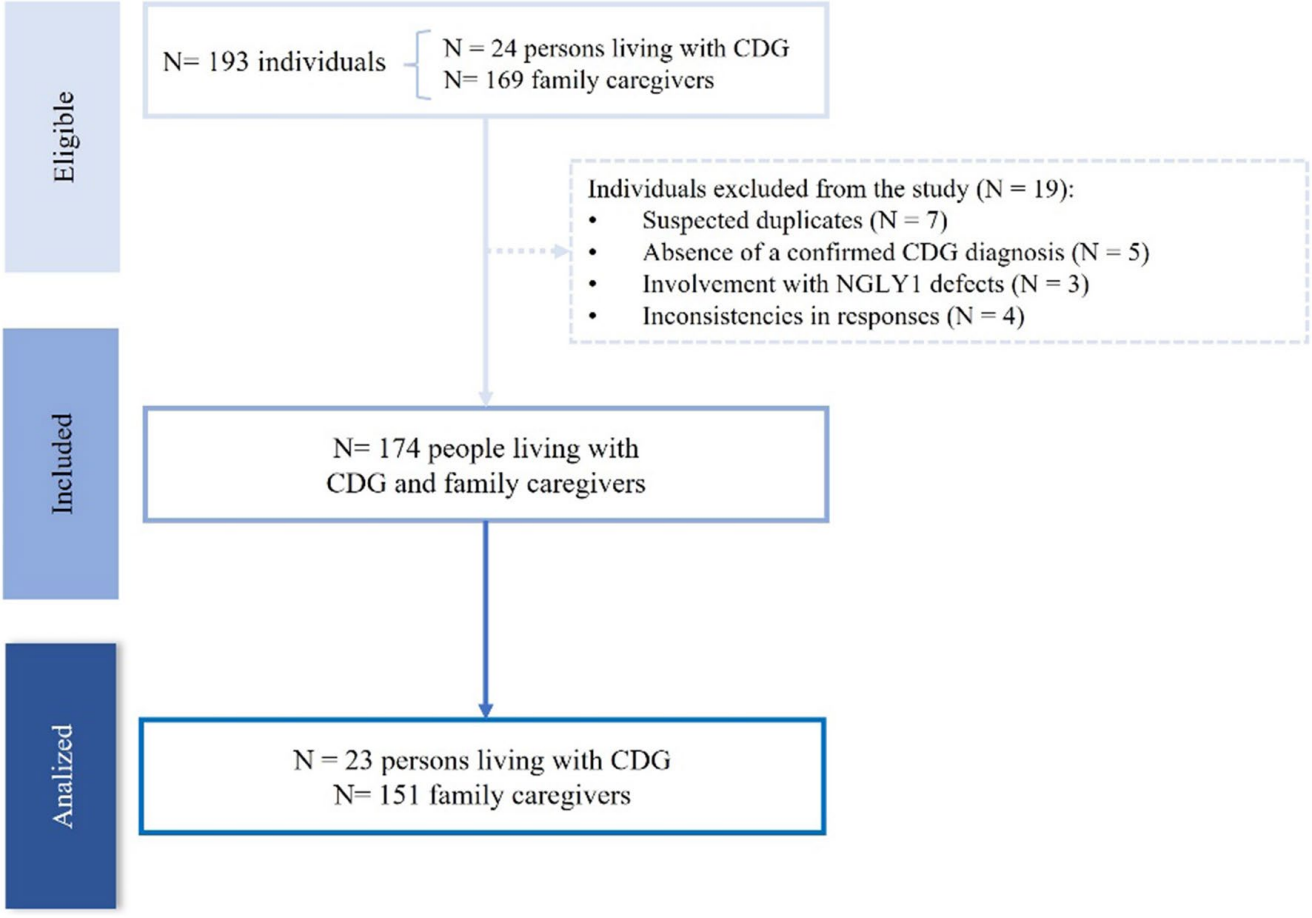
Strobe flowchart on participants eligibility and sample included and analyzed in the study

According to the most recent paper on CDG epidemiology, the number of people living with CDG identified worldwide in 2022 is 3057 [ref]. Despite the identification of 163 genes linked to CDG [33], only 93 CDG cases have available epidemiological data, suggesting that a significant number of CDG cases may still go unreported [ref]. To be eligible, all participants had to be at least 18 years old, and patients must have a confirmatory CDG diagnosis. To be considered a family caregiver, familiars were required to have a relationship with a person living with CDG (e.g., mother, father, sibling, or grandparent) and be involved to some degree in caring for that person. In total, 193 individuals responded to the CDG Journey Mapping Questionnaire (Fig. 1). Nineteen individuals were excluded due to duplicate responses, absence of a confirmed CDG diagnosis, involvement with NGLY1-congenital disorder of deglycosylation (NGLY1-CDDG) and inconsistency in responses (Fig. 1). The sample analyzed in this study included 23 persons living with CDG (13.2%) and 151 family caregivers (86.8%).

### Instruments

*Sociodemographic data* We collected information about participants’ sex, age, marital status, educational degree, and current occupation.

*Information about CDG clinical features, and professional and social support* Both individuals living with CDG and family caregivers were asked to indicate the CDG gene mutation and how long it took to reach a final CDG diagnosis. We also questioned the participants if they were receiving any kind of healthcare professional support (from whom, and/or from which medical specialty) and if they are currently in contact with other families or people with the same or a similar disease (social support).

*The Brief Resilience Coping Scale (BRCS)* The BRCS measures how people cope with stress in an adaptive manner by identifying individuals with lower levels of resilience, aiming to intervene to enhance their resilience coping skills [52]. This scale was created by Sinclair and Wallston (2004), based on a nine-item version, and ended in a final version of four items scale [52]. The themes that emerged from these four items were tenacity, optimism, creativity, an aggressive approach to problem-solving, and a commitment to extract positive growth from difficult situations [52]. Each item was answered based on a 5-point Likert scale, from “Does not describe you at all” to “It describes you very well”. The sum of the scores ranged from 4 to 20. Low-resilient copers scored 13 or less, medium-resilient individuals scored between 14 and 17, and high-resilient individuals scored more than 17 [52]. An exploratory factorial analysis performed with the total sample (N = 174) confirmed the adequacy of the scale (Kaiser–Meyer–Olkin, KMO = 0.772; Bartlett’s test of sphericity χ^2^_(6)_ = 145.446, *p* < 0.001) and the existence of one factor including all items, which explained 59.15% of the total variance (factor loadings between 0.735 and 0.789). The scale also presented a good internal consistency (Ω = 0.768) and adequate inter-item correlation mean (IICM = 0.455) and item-total correlations (between 0.525 and 0.596).

### Procedure

The study received ethical approval from the Faculty of Psychology at the University of Lisbon and all participants provided informed consent electronically. The online e-questionnaire was disseminated through various channels, including direct messaging via email, WhatsApp, Facebook, and Twitter, and remained active from May 15, 2021, to October 15, 2021.

### Statistical analysis

We used the software package IBM SPSS Statistics for Windows, version 27.0, to perform all statistical analyses. Descriptive analyses were performed to characterize the samples of persons living with CDG and family caregivers. We run Spearman coefficient to assess correlations between resilience and specific variables (i.e., age, academic degree, time until a final CDG diagnosis) in each sample. Independent t-tests were also used to compare resilience scores between patients and family caregivers’ samples or between other independent groups (i.e., sex, marital status, occupation, contact with other families/persons with the same or a similar CDG, receiving professional support regarding CDG condition).

## Results

### Sample characteristics: sociodemographic data, CDG clinical features, professional and social support, and resilient coping.

Table 1 presents the results regarding the sociodemographic characteristics of the two samples. Most participants were female between 25 and 54 years old (person living with CDG: 86.9%, family caregivers: 88.0%) and were married or living in a life-in partnership. Most family caregivers had a higher academic degree (78.2%), but only 47.8% of the persons living with CDG had a high degree or equivalent. Although most participants were employed or self-employed, a relevant percentage of persons with CDG were unemployed and unable to work due to their health condition (21.7%).

**Table 1:** Sociodemographic characteristics of the participants

Sociodemographic characteristics	Person living with CDG (*N* = 23)		Family caregiver (*N* = 151)	
	*n*	*%*	*n*	*%*
Sex				
Male	7	30.4	16	10.6
Female	16	69.6	135	89.4
Age (years)				
25–34	7	30.4	28	18.5
35–44	6	26.1	68	45.0
45–54	7	30.4	37	24.5
55–65	1	4.3	14	9.3
above 65	2	8.7	4	2.7
Marital status				
Single	7	30.4	6	4.0
Married/Live-in partnership	13	56.5	132	87.4
In a relationship	1	4.3	2	1.3
Divorced/Separated	2	8.7	11	7.3
Educational degree				
Less than high school diploma	2	8.7	3	2.0
High school diploma or equivalent degree	11	47.8	28	18.5
Higher academic degree	10	43.4	118	78.2
Other	0	0	2	1.3
Occupation				
Employed/Self-employed	9	39.1	102	67.5
Unemployed and able to work	2	8.7	3	2.0
Unemployed and not able to work (disability, long-term illness)	5	21.7	0	0.0
Homemaker/Houseworker/Full caregiver	1	4.3	32	21.2
Retired	2	8.7	8	5.3
Other	4	17.3	6	4.0

Table 2 shows CDG clinical characterization, time until a final diagnosis, and professional and social support received. The most frequent CDG among the participants living with CDG was GNE myopathy, followed by PMM2-CDG. Additionally, one participant with DDOST- and one with PIGN-CDG were included in the ‘Other' CDG category. Approximately, half of the family caregivers had relatives with PMM2-CDG; other CDG included ALG6-, PIGA-, ALG1-, and PIGN-CDG. Most participants received a final diagnosis of CDG up to 3 years (persons living with CDG: 69.5%, family caregivers: 74.1%). A higher percentage of family caregivers reported having professional support (44.4%) compared with those living with CDG (30.4%). Several healthcare specialties delivered this support. Most persons with CDG receiving professional support were followed by a psychologist (71.4%). Regarding family caregivers, 28.4% were followed by a psychologist, 26.9% by a genetic counselor, 19.4% by a nutritionist or dietitian, and 17.9% by a social worker or a psychiatrist. A large proportion of participants were in contact with other families or people with the same or similar disease.

**Table 2:** Information about participants’ CDG genotype, time until a final diagnosis, and professional and social support

	Person living with CDG *(N* = *23)*		Family caregiver *(N* = *151)*	
	*n*	%	*n*	%
CDG genotype				
PMM2-CDG	7	30.4	82	54.3
GNE myopathy	14	60.8	0	0.0
Other	2	8.8	69	45.7
Time until a final CDG diagnosis				
< 3 months	3	13.0	17	11.3
3–6 months	3	13.0	23	15.2
7–12 months	4	17.4	28	18.5
1–3 years	6	26.1	44	29.1
4–5 years	2	8.7	12	8.0
6–9 years	0	0	8	5.3
10–20 years	0	0	11	7.3
> 20 years	4	17.4	8	5.3
I don’t know	1	4.3	0	0.0
Receive professional support (healthcare or other)				
Yes	7	30.4	67	44.4
No	16	69.6	84	55.6
Contact with other families with a similar disease (CDG)				
Yes	20	87.0	128	84.8
No	3	13.0	23	15.2

Table 3 presents the resilience mean values and classification for individuals living with CDG and family caregivers, indicating that, overall, both groups have medium to high resilience coping levels.

**Table 3:** Means, standard deviations, and classification (low, medium, and high resilient coping) of participants’ resilience levels measured with the BRCS

	Person living with CDG *(N* = *23)*		Family caregiver *(N* = *151)*	
	M	SD	M	SD
BRCS	16.04	2.42	15.10	2.81
	*n*	%	*n*	%
BRCS classification				
Low resilient coping (4–13)	3	13.0	42	27.8
Medium resilient coping (14–16)	10	43.5	66	43.7
High resilient coping (17–20)	10	43.5	43	28.5

### Resilient coping: correlations and group comparisons considering sociodemographic and clinical data, and information about professional and social support

#### Sociodemographic characteristics

Persons with CDG had a higher resilience mean value than family caregivers, although the difference was not statistically significant (T_(172)_ = 1.525, *p* = 0.129). No significant differences regarding resilient coping mean scores were found between males and females, both in persons with CDG (T_(21)_ = − 1.396, *p* = 0.117) and family caregivers (T_(149)_ = 0.038, *p* = 0.969). Concerning the marital and employment status, we compared individuals who are single with those currently in marital or nonmarital romantic relationships and participants who were currently employed with those who were not. We did not find significant resilience differences between these groups for both persons living with CDG (Marital status: T_(21)_ = − 0.105, *p* = 0.917; Occupation: T_(21)_ = − 0.074, *p* = 0.942) and family caregivers (Marital status: T_(149)_ = − 1.220, *p* = 0.224; Occupation: T_(149)_ = 0.794, *p* = 0.428). Furthermore, the relationship between resilient coping and age was not statistically significant in either samples (persons with CDG: r_s_ = .− 0.023, *p* = 0.918; family caregivers: r_s_ = 0.104, *p* = 0.205). Conversely, a positive, strong, and significant correlation was found between academic degree and resilience scores among persons with CDG, suggesting that those with higher educational degree tend to report higher resilience (r_s_ = 0.448, *p* = 0.032); the same conclusion cannot be drawn for family caregivers (r_s_ = − 0.010, *p* = 0.905).

#### CDG genotype and time until a final CDG diagnosis

We compared the resilience mean scores between persons with GNE myopathy and PMM2-CDG or other CDG, based on the different impacts of the disease on motor skills and cognitive development. We found that persons with GNE myopathy had significantly higher resilience scores compared with the remaining persons with CDG (T_(21)_ = − 2.174, *p* = 0.041; GNE myopathy: M = 16.86, SD = 1.90; other CDG: M = 14.78, SD = 2.73).

We did not find significant correlations between the time until a final CDG diagnosis and resilience coping scores for both persons living with CDG (r_s_ = 0.192, *p* = 0.379) and their family caregivers (r_s_ = − 0.133, *p* = 0.104). Similarly, the correlation between resilience and time until a final diagnosis was not statistically significant when we run the analysis for persons with GNE myopathy (r_s_ = 0.203, *p* = 0.487) and those with other CDG (r_s_ = 0.052, *p* = 0.895) separately.

#### Professional and social support

The resilience scores for each participant’s sample were compared considering i) whether individuals were receiving professional support regarding their CDG condition (any support vs. no support) and ii) whether individuals were currently in contact with other families or persons with the same or a similar CDG (yes vs. no). We found that family caregivers caring for individuals with CDG had marginally significant higher scores of resilience coping if they were receiving any kind of professional support compared with those who have not (T_(149)_ = − 1.899, *p* = 0.059; any support M = 15.58, SD = 2.66; no support M = 14.71; SD = 2.89). No significant differences were found between persons with CDG who received professional support with those unsupported (T_(7.964)_ = − 0.718, *p* = 0.493).

Moreover, family caregivers who reported having contact with other families or people with the same or similar disease also had marginally significantly higher resilience coping scores compared with those who did not (T_(149)_ = 1.973, *p* = 0.050; contact M = 15.29, SD = 2.77; no contact M = 14.04; SD = 2.87). This statistical analysis was not performed for persons with CDG, since almost all had contact with other families and/or persons with similar health conditions.

## Discussion

Resilience directly shapes how people live and, importantly, can impact the health status and the capacity to face a life-threatening disease. The main goal of this study was to measure the levels of resilience in patients and family caregivers living with CDG, using the BRCS. In addition, we aimed to explore if and how resilience scores change considering participants` sociodemographic characteristics, CDG clinical features, and professional and social support.

Our results showed that most persons living with CDG and family caregivers exhibited medium or high resilience levels, which corroborate with previous findings reported for other severe diseases [15, 18, 19, 53, 54]. Although the disease can have a different impact on patients and family caregivers’ lives, these are encouraging results as both seem to maintain interesting levels of tenacity and resilience, despite the demanding challenges and complexities of living with this serious condition. This research also showed that people with GNE myopathy had significantly higher resiliency than those with PMM2-CDG or other CDG. GNE myopathy is a physical disorder characterized by muscle weakness and atrophy with later onset (e.g., usually during early adulthood) [53]. On the other hand, PMM2-CDG presents early onset manifestations with frequently severe neurological disability [54]. The late onset and the absence of neurological impairment in GNE myopathy individuals could affect less their coping skills and overall well-being, resulting in higher resilience behaviors.

The positive significant correlation between resilience coping scores and CDG patients´ educational degree is also consistent with previous investigations [55, 56]. It is well-reported that more educated people are better prepared to face challenges and have better psychosocial health than less educated people [57]. For example, in a study carried out with patients with sickle cell disease (a genetic disorder causing anemia and intense episodes of extreme pain), the authors demonstrated a strong correlation between education level and psychological resilience [58]. Furthermore, research in patients with chronic kidney disease revealed that factors affecting the resilience of the high-risk group included level of education and health-promoting behaviors [59]. Nevertheless, it is noteworthy to underlie that most patients in this study have GNE myopathy, a rare condition devoid of neurological dysfunction, which enables educational progression, compared with other severe forms of CDG (e.g., PMM2-CDG).

Regarding family caregivers, we saw marginally significant higher levels of resilience for those who received professional and social support compared with unsupported individuals. Research on caregivers caring for patients with dementia has demonstrated that the access and acceptance of professional support are associated with decreased burden, increased satisfaction [60] and well-being [61], and self-efficacy in managing problem behaviors [62], promoting caregiver resilience [63]. Similarly, Spence et al. (2008) concluded that family caregivers caring for patients with advanced chronic obstructive pulmonary disease need professional (and social) support to ensure their physical and emotional health and to promote resilience [64]. Usually, in rare diseases, professional support starts with physicians providing general medical information to family caregivers regarding the disease, treatment options and conceptualizing life changes and implications for the future [65]. However, given the severe impairment of overall patients’ functioning provoked by these diseases and the expected burden on families in the long term, it is imperative to provide life-long multidisciplinary healthcare support for both patients and caregivers [66]. In our study, we found a high percentage of participants who reported not having any professional healthcare support. Although the data were collected in several countries, and the reason for not being accompanied by a professional was not determined, we understand that this may be a worrying sign of lack of available resources (both human and financial) to access this support. In this sense, health institutions and patient associations must continue to make efforts to assess the needs and support patients with CDG and their families throughout their lives.

Regarding social support, several studies on chronic diseases have showed an association between overall social support and family caregivers’ resilience [67, 68]. People with developed social networks tend to use more positive coping strategies and have lower levels of depression [69]. In addition, Wilks and co-workers (2008) evidenced that support from family and friends as external resources may function as a protective factor of resilience [70]. Other study remarked that caregivers who have more family support were more prone to be resilient and have higher QoL [71]. Rare diseases patients’ organizations (POs) also can empower family caregivers by building a community and connecting parents of affected children with common diagnoses or similar diseases with peers, healthcare professionals, and researchers, enhancing social and professional support [72–74]. POs can also play a crucial role by promoting health literacy [75, 76], raising research funding, and speed up patient access to therapeutic care [75]. The CDG community can obtain informational support related to research, clinical trials, treatment options, epidemiology, care, and other areas on websites like CDG Hub [77], CDG Care [78], World CDG Organization [84], and ClinicalTrials.gov [80]. The World CDG Organization website also includes a mental health section with toolkits and guides to help people living with CDG and family caregivers develop coping strategies to increase their resilience levels.

## Limitations and strengths

The inherent difficulties associated with recruiting participants for studies on rare diseases and the high heterogeneity of CDG might compromise the representativeness of samples, impairing the generalization of the results obtained. It is essential to recognize that CDG is a rare condition usually characterized by severe clinical manifestations, including high neurological commitment, which limits the participation of many patients in this type of research. In our study, only few persons living with CDG answered the questionnaire; most patients were diagnosed with GNE myopathy, which is a physical disorder with different onset and less severe symptomatology compared to other CDG. Partly due to this challenging context, this work was not exhaustive on examining other factors that might explain resilience variability. Persons with the same CDG type may differ significantly regarding their disability degree and experience a wide variation of the disease severity across time. Specific aspects related to the patient's journey (e.g., experiences regarding diagnosis disclosure), or stage of disease when participating in the study were also not investigated.

Nonetheless, our study also has strengths. This is the first research that explored the levels of resilience in patients and family caregivers living with CDG worldwide and explore its relationships with sociodemographic characteristics, CDG clinical features, and professional and social support. Therefore, the study’s originality might motivate future research on mental health in the rare disease field. Moreover, we used a well-validated and reliable psychometric scale to assess resiliency in CDG patients and family caregivers. This scale is particularly useful for people who have difficulty completing longer questionnaires, as happens with CDG patients. These results encourage us to continue to study the mechanisms and coping skills that allow the maintenance of moderate levels of resilience in relatives and patients with CDG in more and less aggressive phenotypes. On the other hand, it will be relevant to understand which fringes of the CDG community the resilience scores are lower to assess the needs and develop specific intervention programs.

## Conclusions

This study concluded that most persons living with CDG and family caregivers showed medium to high resilience levels, despite the inherited difficulties of living with a life-threatening disease. These are positive and encouraging results, considering the high diversity and severity of CDG clinical manifestations, lack of information, insufficient treatment options, and taking into consideration the challenging journey to get a final and accurate diagnosis. Persons living with GNE myopathy have a milder form of the disease, compared with other CDG, which can contribute to reducing stress factors that jeopardize their resilience, allowing them to feel more positive and confident about the future. On the other hand, the professional and social support that family caregivers receive can strengthen their abilities to cope with the challenging demands of caring for people living with CDG, increasing or not diminishing their levels of resilience. These findings gave a first exploratory overview of how resilience scores change in individuals with different characteristics and receiving or not professional and social support. This is extremely useful to develop further research in vulnerable communities with severe forms of illness and develop appropriate interventions and training programs to help the CDG community increase their coping skills and considerably improve their well-being and QoL.

## Data Availability

Not applicable.

## Abbreviations

BRCS: Brief resilience coping scale
CDG: Congenital disorders of glycosylation
GNE: UDP-N-acetylglucosamine 2-epimerase/N-acetylmannosamine kinase
HRQoL: Health-related quality of life
PMM2: Phosphomannomutase deficiency
PO: Patient organizations
QoL: Quality of life
